# The dose can make the poison: lessons learned from adverse *in vivo *toxicities caused by RNAi overexpression

**DOI:** 10.1186/1758-907X-2-8

**Published:** 2011-10-26

**Authors:** Dirk Grimm

**Affiliations:** 1 Cluster of Excellence CellNetworks, Department of Infectious Diseases, Virology, University of Heidelberg, BioQuant BQ0030, Room 502a, Im Neuenheimer Feld 267, D-69120 Heidelberg, Germany

**Keywords:** RNAi, shRNA, miRNA, viral vector, toxicity, saturation

## Abstract

For the past five years, evidence has accumulated that vector-mediated robust RNA interference (RNAi) expression can trigger severe side effects in small and large animals, from cytotoxicity and accelerated tumorigenesis to organ failure and death. The recurring notions in these studies that a critical parameter is the strength of RNAi expression and that Exportin-5 and the Argonaute proteins are rate-limiting mammalian RNAi, strongly imply dose-dependent saturation of the endogenous miRNA pathway as one of the underlying mechanisms. This minireview summarizes the relevant work and data leading to this intriguing model and highlights potential avenues by which to alleviate RNAi-induced toxicities in future clinical applications.

## Background

Since the seminal 1998 report of RNA interference (RNAi) in nematodes [1], the ascent of RNAi technologies from a curious phenomenon in worms to a widely and routinely used surrogate genetic tool in higher eukaryotes, as well as one of our most promising therapeutic modalities, has been nothing short of meteoric. Ironically, though, in the same year, 2006, that the rise of RNAi temporarily culminated in the Nobel Prize for its pioneers Andrew Fire and Craig Mello, Mark Kay's group published a startling study reporting fatal side effects from abundant RNAi expression in the livers of adult mice [2]. Since then a series of further studies in various species and tissues have solidified the original idea that one crucial mechanism underlying the observed *in vivo *toxicities or fatalities is adverse saturation of the endogenous miRNA machinery by ectopic RNAi triggers. Herein I briefly review these papers and findings before highlighting key lessons that we can learn and new avenues that we can now take.

### From observations of dose-dependent *in vivo *RNAi toxicities…

The 2006 Grimm *et al. *study [2] came as a surprise to the field, as the wealth of previous reports had proved RNAi's superior efficacy and thus fostered a rapid translation of RNAi technologies from bench to bedside. What was so different in this particular work was the unique combination of (1) an utmost potent viral RNAi delivery vector (self-complementary adeno-associated virus serotype 8 (scAAV8)), (2) a powerful promoter (U6, one of the strongest known RNA polymerase III promoters) driving small hairpin RNA (shRNA) expression and (3) delivery of high vector doses (directly into the hepatic circulation in some animals) [2]. This experimental setup not only ensured complete liver transduction in the injected mice but also introduced, on average, thousand RNAi expression templates into each hepatocyte, likely resulting in the transcription of hundreds of thousands of shRNA molecules per cell.

Unsurprisingly, at least in retrospect, such massive overloading of the cells with exogenous RNAi inducers was most likely more than what their endogenous RNAi machinery could handle. This is evidenced by the study's finding that more than 20 different abundantly expressed shRNAs caused substantial hepatotoxicities and eventual fatalities, regardless of the presence or absence of targets and without other detectable adverse reactions, such as immune responses. Notably, shRNA overexpression and toxicity correlated with dysregulation of hepatocellular miRNAs, implying competition of shRNAs and miRNAs for rate-limiting factors and substantiating the idea that saturation of the liver RNAi machinery was a major cause of toxicity.

Subsequently, a series of other studies made very similar observations in mouse livers and came to a comparable conclusion. This includes a recent report by Borel and colleagues [3], who also used scAAV8 for *in vivo *shRNA transduction and noted viral dose-dependent hepatotoxicities in mice, which were evidenced by increases in plasma transaminases and animal weight loss and culminated in one death. Furthermore, they also observed shRNA-dependent downregulation of three cellular miRNAs, including liver-specific miR-122, corroborating that shRNA overexpression can adversely perturb the miRNA/RNAi machinery *in vivo*. In line with this evidence, Ahn *et al. *[4] noted gradual hepatocyte death in mice treated with shRNA-expressing gutless adenoviral vectors that correlated with the buildup of mature shRNA molecules and the upregulation of miRNA-controlled hepatic genes.

Researchers who have studied organs other than the liver have described similar notions of shRNA-associated toxicities in the central nervous system (CNS) of the mouse and rat. Notable examples came from Beverly Davidson's group [5], who expressed three shRNAs plus a control against the Huntington's disease homolog in mouse striatum and observed significant neurotoxicities with two active shRNAs as well as the control shRNA. All shRNAs were expressed from the potent U6 promoter and were delivered via efficient AAV1 vectors, and toxicity correlated with shRNA abundance but not with silencing activity. In a later study, the same group again noted severe neurotoxicity with another AAV/U6-driven shRNA, this time in mouse cerebellum [6]. These conditions and findings are highly reminiscent of those in the Grimm *et al. *study in the liver [2], implying that nonspecific shRNA toxicity can occur in multiple cell and tissue types *in vivo*. Indeed, Martin *et al. *[7] recently inadvertently recapitulated shRNA-induced lethalities using AAV1/U6 vectors to express three distinct shRNAs in the striata of various mouse strains and validated the evidence for the involvement of shRNA-induced miRNA dysregulation.

Exemplifying that shRNA toxicity in the CNS is not species-specific, Ulusoy *et al. *[8] reported cytotoxicity from shRNA overexpression in the rat substantia nigra. Using AAV5 to deliver two shRNAs against tyrosine hydroxylase plus two controls, that group noted a dose-dependent loss of nigral dopaminergic neurons with all four shRNAs. Similarly, Khodr and co-workers [9] reported neuron loss in the substantia nigra of rats injected with AAV2 expressing an shRNA against α-synuclein or an irrelevant control shRNA. Moreover, Ehlert *et al. *[10] found a dose-dependent adverse tissue response and neuronal degeneration following AAV1-mediated expression of three distinct shRNAs (including one control) in the red nucleus of rats. Lowering virus amounts, and thus shRNA expression, reduced these effects, and toxicity was absent when an inferior (as compared to AAV1) AAV5 vector was used in another cell type, together providing further support for the saturation model.

Next to rodent liver and CNS, Bish *et al. *[11] recently reported severe cardiac dysfunction and toxicity in three dogs treated with scAAV6-expressing anti-phospholamban shRNA. Their finding that shRNA treatment is associated with alterations in expression of two cellular miRNAs suggests that toxic oversaturation of endogenous RNAi pathways can also occur in large animals.

### …to first insights into the underlying cellular mechanisms…

As noted, the recurrent correlations of cytotoxicities with shRNA abundance and miRNA dysregulation in many reports fuel the model that ectopic shRNA expression can saturate key factors in the miRNA processing pathway. Ample support for this concept is actually provided by numerous studies recapitulating the *in vivo *findings in cultured cells. For instance, the Chen group [12] showed that high-level, U6-driven shRNA expression from a lentiviral vector causes cytotoxicity in primary human lymphocytes, which could be relieved by encoding the same shRNA under the weaker H1 promoter. Likewise, Pan *et al. *[13] noted downregulation of hepatic miRNAs in a liver cell line infected with U6-shRNA-encoding lentiviral vectors. Moreover, Khan *et al. *[14] conducted an extensive meta-analysis of over 150 siRNA or shRNA transfection experiments in which they described frequent upregulation of miRNA-controlled genes upon abundant siRNA and/or shRNA expression. Importantly, some of these studies also provide clues to the limiting RNAi factors. Thus far the leading suspects that have emerged from this *in vitro *work are Exportin-5, the nuclear karyopherin that shuttles shRNAs and miRNAs into the cytoplasm, as well as Argonaute-2, a critical RNA-induced silencing complex (RISC) component that binds and cleaves targeted mRNAs [2,15-18]. Interestingly, Bennasser and colleagues [19] recently reported that Exportin-5 saturation may also reduce Dicer expression and hence activity, adding another layer of complexity to the cellular mechanisms underlying RNAi toxicity.

Validating the potential rate-limiting nature of these factors in an *in vivo *setting is obviously more challenging, yet early reports are rapidly accumulating. In fact, hepatic Exportin-5 and Argonaute-2 coexpression from AAV vectors was recently shown to increase shRNA potency in the livers of adult mice and to partly alleviate RNAi toxicity, implying that these two factors are also prone to exogenous saturation *in vivo *[2,17]. There is further indirect support in a recent study [7] for a correlation of diminished Exportin-5 levels in a particular mouse strain with an increased susceptibility to shRNA-induced neurotoxicity, and others have proposed that the relatively low Exportin-5 expression in the brain may generally render this organ particularly sensitive to adverse saturation effects [10]. Notably, despite the absence of reports to date on *in vivo *morbidities or deaths resulting from siRNA delivery, there is clear evidence that high intracellular siRNA abundance can also saturate critical RNAi components. For instance, the Rossi group [15] reported that transfected siRNAs can compete with each other, with cotransfected shRNAs or with endogenous miRNAs for RISC incorporation. As with shRNAs, Argonaute-2 appears to play a crucial role in this process, as its overexpression has been shown to at least partially relieve some of these competition effects [17]. Moreover, as noted above, Khan *et al. *[14] found that siRNA transfection frequently perturbs cellular miRNA expression and thus regulation of endogenous gene expression. These and additional similar findings clearly imply that dose- and saturation-dependent cytotoxicity is not restricted to vector-encoded shRNAs, but can be induced and observed with siRNA delivery. That more severe effects still have not been noted *in vivo *may be related to the facts that (1) achieving high intracellular doses of siRNAs is difficult compared to shRNA expression and (2) the typically short-term kinetics of siRNA persistence and activity may not suffice to perturb the cellular RNAi machinery to an extent that would cause toxicity.

### ...and to novel clinically relevant strategies to alleviate RNAi toxicity

**Figure 1:**
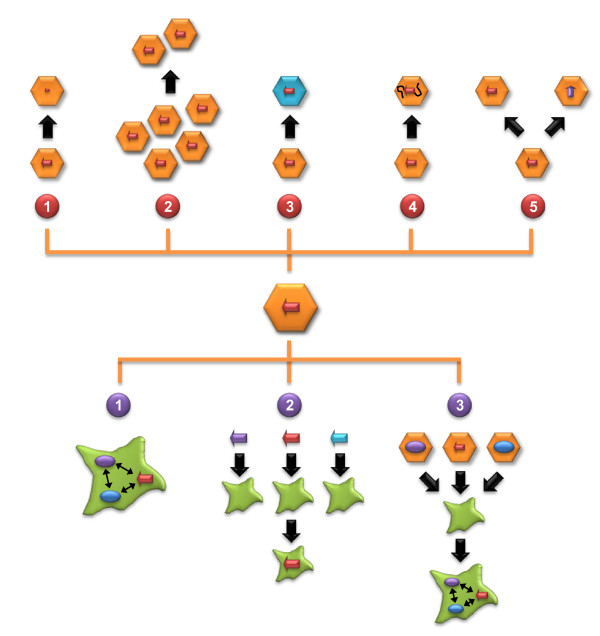
**Schematic overview of strategies to alleviate *in vivo *RNAi toxicity**. As explained in the text, one can roughly distinguish strategies that aim to improve the RNA interference (RNAi) vector itself (top half) or that instead focus on the cellular component (bottom half). Amongst the former, the main approaches reported so far include a reduction of short hairpin RNA (shRNA) expression per given vector dose via the use of moderate promoters (1-red), a limitation of the overall vector dose applied (2-red), shRNA delivery via a specific viral vector serotype (3-red), embedding of the shRNA sequence into a miRNA scaffold (4-red) or a combination of RNAi technologies with further inhibitors of gene expression (5-red). On the cellular side, currently pursued strategies comprise mathematical modeling of all rate-limiting factors in the cell (1-purple), cell-based screening for potent and minimally toxic RNAi triggers (2-purple) and controlled overexpression of known rate-limiting cellular components, together with the RNAi vector (3-purple).

The available evidence to date suggests that a major goal for future clinical RNAi applications must be to thwart the risk of saturating endogenous RNAi pathways by exogenous shRNAs without compromising their therapeutic efficacy. Toward this aim, a multitude of concepts can be envisioned that fall roughly into two categories: improvements in the RNAi vector itself or advances in our understanding of cellular RNAi mechanisms (Figure 1).

The first category comprises various practical strategies that can be adopted to limit intracellular steady-state levels of ectopic RNAi substrates to tolerable yet still effective degrees. Accordingly, one approach is the use of weaker shRNA promoters, such as H1 or 7SK instead of U6, or moderate and/or tissue-specific RNA polymerase II promoters [17,20]. Lowering vector doses or switching to a less potent viral variant can likewise reduce shRNA expression to safer levels, especially since the therapeutic window for shRNA expression appears to be relatively wide (at least for potent molecules), frequently allowing variations in vector doses of more than two orders of magnitude without major losses in target knockdown activities [2,8,10,17]. However, for some applications, lowering the vector doses below a critical threshold may adversely interfere with a need for complete target tissue transduction, implying that this avenue will have to be adapted to each scenario [6,10]. Another useful strategy may be to express active RNAi sequences from a miRNA scaffold, which has proved particularly valuable in the CNS thus far [5,6]. The reasons for the presumably lower *in vivo *toxicity from miRNA-like vectors as compared to shRNA counterparts are still not fully clear, but the generally reduced abundance of mature miRNA strands may play a critical role. Also, since miRNAs are being shunted through an endogenous processing pathway, their entry into RISC might be slower compared to highly expressed shRNAs or transfected siRNAs. It has been proposed that this may in turn further reduce the likelihood of adverse saturation effects [15]. On the other hand, since miRNA biogenesis already begins with Drosha processing (a step that is skipped by siRNAs and shRNAs), an important question for future research will be to investigate the possibility of specific nuclear saturation events with miRNA vectors. Combining low-copy RNAi strategies with other modes of gene silencing, such as U1 inhibition or ribozymes, can also maintain high efficiency while minimizing saturation risks [21-23].

Regarding the second category, several laboratories have initiated attempts to mathematically model RNAi in mammalian cells [24,25], which will hopefully further help researchers to rationally improve the efficacy and safety of their RNAi strategies. For instance, Cuccato *et al. *[24] calculated the number of active RISC to be in a range from 10^3 ^to 10^4 ^in a typical mammalian cell and accordingly proposed that the number of ectopic RNAi triggers ideally be kept below this range to avoid saturation effects. Likewise, Arvey *et al. *[25] presented a mathematical model according to which the efficiency of therapeutic RNAi molecules on their intended target depends on the overall abundance of potential binding sites in the cell. Hence an important implication is that to prevent this dilution effect and to maximize siRNA or shRNA potency, sequences that have minimal off-targets should be preferred. This will not only eliminate unintended silencing effects but also permit decreasing the dose of the RNAi trigger, which will in turn minimize the risk of nonspecific saturation effects.

In addition, other investigators are concomitantly screening libraries of RNAi inducers in a high-throughput fashion for potent and safe molecules [26]. As with the modeling approach, their aim is to identify favorable features of RNAi triggers and unravel rate-limiting cellular steps and components to ultimately deduce guidelines for the rational design of optimized RNAi templates and strategies. Most critical here will be the use of appropriate model systems which take into consideration that the manifestation and extent of RNAi-associated toxicities can vary greatly with the specific cell type and depend on many other variables that may not be easy to truly mimic *in vitro*. An example of the latter is the observation by Beer *et al. *[27] that even low doses of vector-encoded shRNAs can have fatal side effects in mice that coexpress a proto-oncogene in the liver. In these animals, even marginal hepatocyte death was sufficient to accelerate tumorigenesis, a phenomenon that would have been missed in isolated cell cultures. If, however, such cultures have to be used, it may be important to employ primary human cells (where available) instead of established cell lines. This is implied by the study by An *et al. *[12], for instance, wherein shRNA cytotoxicity manifested only in primary lymphocytes and not in a human T-cell line. Also notable along these lines are data published by Martin *et al. *[7] indicating that the genetic background of rodents can modify their sensitivity to RNAi toxicity, together suggesting that selecting proper cell types and animal strains is a very important consideration in preclinical RNAi trials.

The aforementioned strategy to deliberately coexpress known rate-limiting cellular RNAi factors can boost shRNA potency and reduce toxicity [2,17], yet the long-term outcomes of this particular approach for the cell and organism remain to be studied. It is interesting to note in this context that a series of recent findings have indicated that essential parts of the RNAi machinery are inherently dysregulated in many cancers or during infections with viral pathogens [28]. This suggests that, along with the mathematical strategies described above, an important goal for future (pre-)clinical research should be quantitative delineation of the exact concentrations of all RNAi components in a given cell, of the intrinsic and extrinsic silencing triggers and of their target mRNAs so that researchers can become able to adapt and fine-tune therapeutic strategies toward maximum efficiency and minimum toxicity.

## Conclusion

Regarding the pace at which the field has moved from the first notion of *in vivo *RNAi toxicities in 2006 to today's wealth of novel options and innovative concepts to alleviate these toxicities, and considering the rapidly increasing numbers of studies reporting the successful implementation of these avenues in animals, we can certainly stay highly optimistic that the realization of safe and potent RNAi strategies in humans remains a most realistic goal for the near future.

## Abbreviations

AAV: adeno-associated virus; CNS: central nervous system; miRNA: microRNA; RNAi: RNA interference; scAAV: self-complementary adeno-associated virus; shRNA: short hairpin RNA; siRNA: small interfering RNA.

## Competing interests

The author declares that he has no competing interests.
